# Crystal structure of 1,2-di­benzoyl­ace­naphthyl­ene

**DOI:** 10.1107/S2056989015011160

**Published:** 2015-06-13

**Authors:** Fred H. Greenberg, Alexander Y. Nazarenko

**Affiliations:** a Chemistry Department, SUNY Buffalo State, 1300 Elmwood Ave, Buffalo, NY 14222, USA

**Keywords:** crystal structure, 1,2-di­benzoyl­ace­naphthyl­ene, crystal packing

## Abstract

The title mol­ecule, C_26_H_16_O_2_, crystallizes as a mol­ecular crystal with no strong inter­molecular inter­actions (the shortest C—H⋯O contact is longer than 3.4 Å). Two flat ace­naphthyl­ene groups of neigboring 1,2-di­benzoyl­ace­naphthyl­ene mol­ecules are related by a crystallographic center of symmetry and are stacked with the distance between their mean planes of 3.37 (1) Å, apparently making an optimal close packing for these bulky aromatic moieties. Both carbonyl groups are oriented towards the same side of the planar ace­naphthyl­ene. The angles between the flat ace­naphthyl­ene group and the benzoyl groups are 62.6 (1) and 57.8 (1)°. Because rotation of the benzoyl groups is sterically hindered, we expect that the mol­ecules will remain locked in this ‘pseudo-*cis*’ orientation in solution. As a result, reduction of 1,2-di­benzoyl­ace­naphthyl­ene at low temperature with sodium di­thio­nite yields the *cis*-isomer of 1,2-dibenzoyl-1,2-di­hydro­ace­naphthyl­ene, which is sterically favorable. This isomer is thermodynamically less favorable than the *trans* isomer, but it converts to the more stable isomer only on long-term heating (Greenberg & Schenendorf (1980 ▸).

## Related literature   

For synthesis and reactions of the title compound, see: Greenberg & Schenendorf (1980 ▸); Dilthey *et al.* (1938 ▸). For packing in mol­ecular crystals of polyaromatic compounds, see: Kitaigorodsky (1973 ▸).
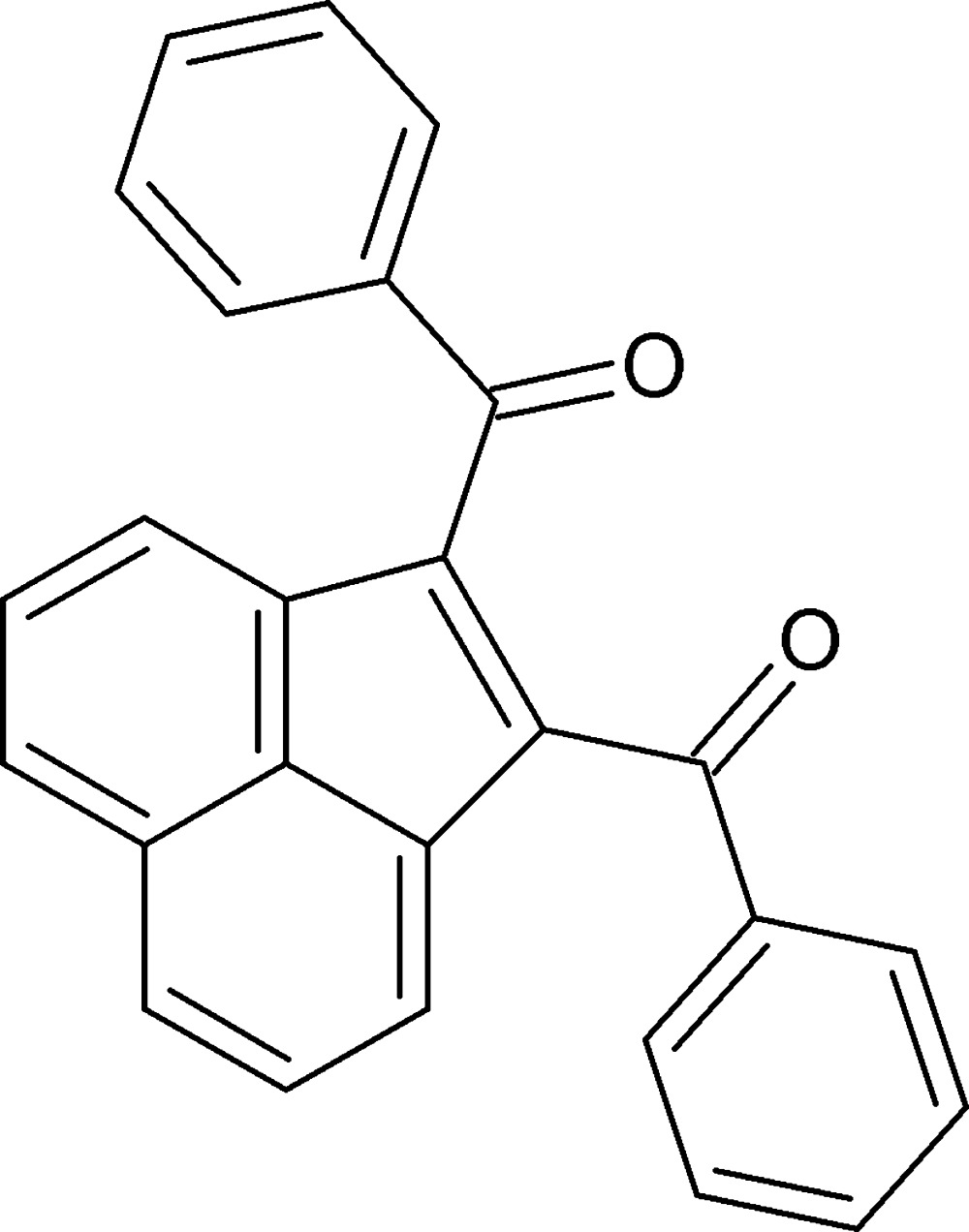


## Experimental   

### Crystal data  

C_26_H_16_O_2_
*M* *_r_* = 360.39
Triclinic, 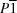

*a* = 9.4578 (4) Å
*b* = 10.2665 (5) Å
*c* = 10.9183 (4) Å
α = 71.448 (2)°
β = 66.494 (2)°
γ = 84.269 (2)°
*V* = 921.21 (7) Å^3^
*Z* = 2
Mo *K*α radiation
μ = 0.08 mm^−1^
*T* = 173 K
0.69 × 0.65 × 0.41 mm

### Data collection  

Bruker PHOTON-100 CMOS diffractometer
Absorption correction: numerical (*SADABS2014*/5; Bruker, 2014 ▸) *T*_min_ = 0.867, *T*_max_ = 0.951
32789 measured reflections
4661 independent reflections
3721 reflections with *I* > 2σ(*I*)
*R*_int_ = 0.044

### Refinement  

*R*[*F* ^2^ > 2σ(*F* ^2^)] = 0.044
*wR*(*F* ^2^) = 0.119
*S* = 1.05
4661 reflections
317 parameters
All H-atom parameters refined
Δρ_max_ = 0.29 e Å^−3^
Δρ_min_ = −0.23 e Å^−3^

### 

Data collection: *APEX2* (Bruker, 2013 ▸); cell refinement: *SAINT* (Bruker, 2013 ▸); data reduction: *SAINT*; program(s) used to solve structure: *SHELXT* (Sheldrick, 2015*b* ▸); program(s) used to refine structure: *SHELXL2014* (Sheldrick, 2015*a* ▸); molecular graphics: *OLEX2* (Dolomanov *et al.*, 2009 ▸); software used to prepare material for publication: *OLEX2*.

## Supplementary Material

Crystal structure: contains datablock(s) I. DOI: 10.1107/S2056989015011160/zl2628sup1.cif

Structure factors: contains datablock(s) I. DOI: 10.1107/S2056989015011160/zl2628Isup2.hkl

Supporting information file. DOI: 10.1107/S2056989015011160/zl2628Isup3.cdx

Supporting information file. DOI: 10.1107/S2056989015011160/zl2628Isup4.txt

Supporting information file. DOI: 10.1107/S2056989015011160/zl2628Isup5.cml

**Fig. 1.**
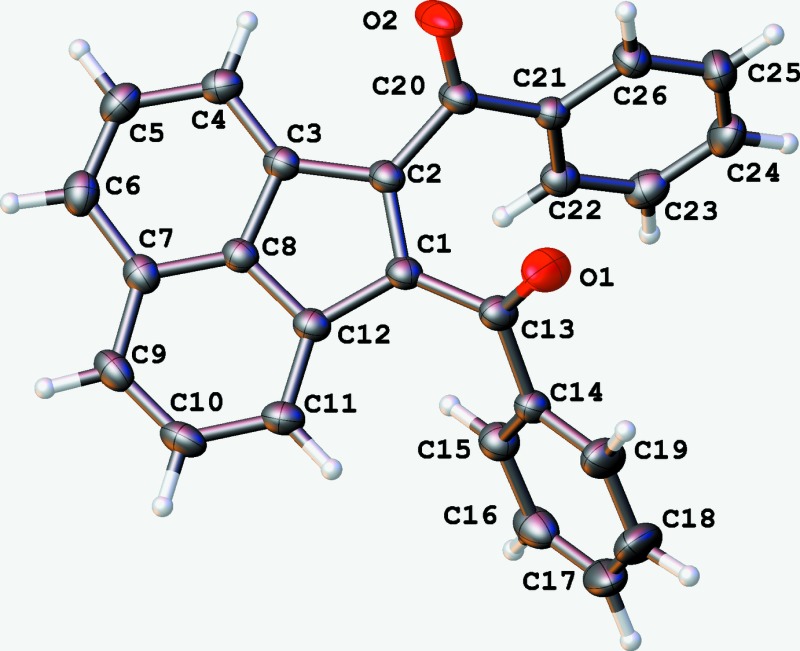
The molecular structure of the title compound. Dispalcement elipsoids are drawn at 50% probability level.

**Fig. 2.**
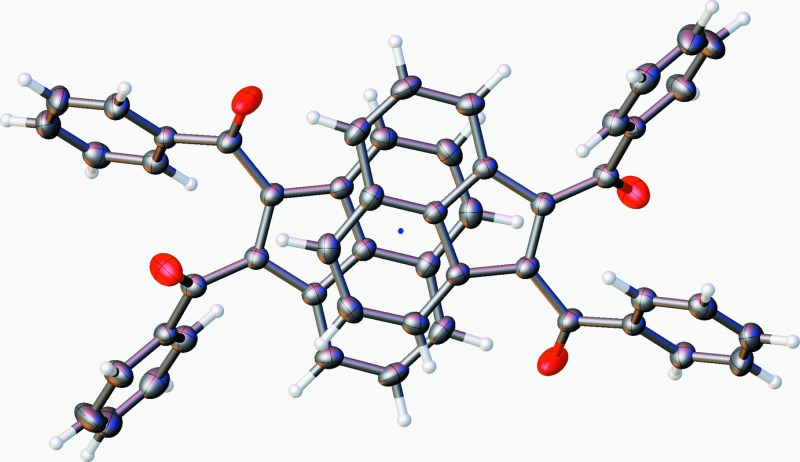
Two "stacked" molecules of the title compound (symmetry operator -x, 1 - y, 2 - z). View along the perpendicular to the mean plane of acenaphthylene ring. The center of symmetry is shown in blue.

. DOI: 10.1107/S2056989015011160/zl2628fig1.tif
The mol­ecular structure of the title compound. Dispalcement elipsoids are drawn at 50% probability level.

x y z . DOI: 10.1107/S2056989015011160/zl2628fig2.tif
Two "stacked" mol­ecules of the title compound (symmetry operator −*x*, 1 − *y*, 2 − *z*). View along the perpendicular to the mean plane of ace­naphthyl­ene ring. The center of symmetry is shown in blue.

CCDC reference: 1405661

Additional supporting information:  crystallographic information; 3D view; checkCIF report

## supplementary crystallographic information

### S1. Synthesis and crystallization

Synthesis of the title compound is described in Greenberg & Schenendorf (1980).

### S2. Refinement

Crystal data, data collection and structure refinement details are summarized in Table 1.

All hydrogen atoms were located in electron difference density Fourier maps and were refined in an isotropic approximation.

### Crystal data

**Table:**

C_26_H_16_O_2_	*Z* = 2
*M**_r_* = 360.39	*F*(000) = 376
Triclinic, *P*1	*D*_x_ = 1.299 Mg m^−^^3^
*a* = 9.4578 (4) Å	Mo *K*α radiation, λ = 0.71073 Å
*b* = 10.2665 (5) Å	Cell parameters from 9865 reflections
*c* = 10.9183 (4) Å	θ = 2.9–30.5°
α = 71.448 (2)°	µ = 0.08 mm^−^^1^
β = 66.494 (2)°	*T* = 173 K
γ = 84.269 (2)°	Block, yellow
*V* = 921.21 (7) Å^3^	0.69 × 0.65 × 0.41 mm

### Data collection

**Table:**

Bruker PHOTON-100 CMOS diffractometer	3721 reflections with *I* > 2σ(*I*)
Radiation source: sealedtube	*R*_int_ = 0.044
φ and ω scans	θ_max_ = 28.5°, θ_min_ = 2.9°
Absorption correction: numerical (*SADABS2014*/5; Bruker, 2014)	*h* = −12→12
*T*_min_ = 0.867, *T*_max_ = 0.951	*k* = −13→13
32789 measured reflections	*l* = −14→14
4661 independent reflections	

### Refinement

**Table:**

Refinement on *F*^2^	Primary atom site location: structure-invariant direct methods
Least-squares matrix: full	Hydrogen site location: difference Fourier map
*R*[*F*^2^ > 2σ(*F*^2^)] = 0.044	All H-atom parameters refined
*wR*(*F*^2^) = 0.119	*w* = 1/[σ^2^(*F*_o_^2^) + (0.060*P*)^2^ + 0.1858*P*] where *P* = (*F*_o_^2^ + 2*F*_c_^2^)/3
*S* = 1.05	(Δ/σ)_max_ < 0.001
4661 reflections	Δρ_max_ = 0.29 e Å^−^^3^
317 parameters	Δρ_min_ = −0.23 e Å^−^^3^
0 restraints	

### Special details

**Table:**

Experimental. SADABS-2014/5 (Bruker,2014) was used for absorption correction. wR2(int) was 0.0679 before and 0.0587 after correction. The Ratio of minimum to maximum transmission is 0.9117. The λ/2 correction factor is 0.00150.
Geometry. All e.s.d.'s (except the e.s.d. in the dihedral angle between two l.s. planes) are estimated using the full covariance matrix. The cell e.s.d.'s are taken into account individually in the estimation of e.s.d.'s in distances, angles and torsion angles; correlations between e.s.d.'s in cell parameters are only used when they are defined by crystal symmetry. An approximate (isotropic) treatment of cell e.s.d.'s is used for estimating e.s.d.'s involving l.s. planes.

### Fractional atomic coordinates and isotropic or equivalent isotropic displacement parameters (Å^2^)

**Table:**

	*x*	*y*	*z*	*U*_iso_*/*U*_eq_	
O2	0.41366 (12)	0.32535 (12)	1.03180 (10)	0.0490 (3)	
O1	0.28034 (14)	0.09696 (10)	0.86494 (10)	0.0479 (3)	
C21	0.56736 (13)	0.25985 (12)	0.83077 (12)	0.0275 (2)	
C8	0.13285 (13)	0.53667 (12)	0.80666 (11)	0.0269 (2)	
C3	0.23865 (13)	0.51645 (12)	0.87207 (12)	0.0272 (2)	
C1	0.25412 (13)	0.33485 (12)	0.78461 (11)	0.0277 (2)	
C12	0.13773 (13)	0.42876 (12)	0.75109 (11)	0.0273 (2)	
C7	0.03120 (13)	0.64398 (12)	0.80421 (12)	0.0300 (3)	
C13	0.28234 (14)	0.19576 (12)	0.76620 (12)	0.0302 (3)	
C2	0.31430 (13)	0.38697 (12)	0.85609 (12)	0.0284 (2)	
C22	0.62779 (14)	0.30751 (13)	0.68478 (12)	0.0305 (3)	
C14	0.30979 (14)	0.17945 (12)	0.62795 (12)	0.0291 (2)	
C4	0.24678 (14)	0.61137 (13)	0.93445 (13)	0.0320 (3)	
C20	0.42982 (14)	0.32278 (13)	0.91594 (12)	0.0309 (3)	
C9	−0.07298 (14)	0.64151 (14)	0.74029 (13)	0.0351 (3)	
C23	0.75990 (15)	0.25202 (14)	0.60792 (14)	0.0367 (3)	
C11	0.03456 (14)	0.42815 (14)	0.69073 (13)	0.0326 (3)	
C5	0.14578 (16)	0.72285 (14)	0.93168 (14)	0.0361 (3)	
C6	0.04105 (15)	0.73975 (13)	0.87001 (13)	0.0355 (3)	
C15	0.36243 (15)	0.28826 (14)	0.50418 (13)	0.0344 (3)	
C26	0.64006 (16)	0.15492 (14)	0.89884 (14)	0.0353 (3)	
C10	−0.06969 (14)	0.53653 (15)	0.68617 (13)	0.0362 (3)	
C24	0.83096 (17)	0.14701 (16)	0.67630 (16)	0.0431 (3)	
C16	0.39353 (17)	0.26702 (18)	0.37632 (15)	0.0443 (3)	
C19	0.28927 (19)	0.04982 (15)	0.62154 (17)	0.0445 (3)	
C25	0.77049 (18)	0.09843 (15)	0.82128 (16)	0.0435 (3)	
C17	0.3715 (2)	0.1387 (2)	0.37195 (18)	0.0557 (4)	
C18	0.3189 (2)	0.03098 (19)	0.4939 (2)	0.0611 (5)	
H22	0.5757 (17)	0.3818 (15)	0.6373 (15)	0.034 (4)*	
H26	0.5971 (18)	0.1216 (15)	1.0037 (16)	0.039 (4)*	
H4	0.3157 (18)	0.6005 (15)	0.9825 (15)	0.039 (4)*	
H9	−0.1493 (18)	0.7137 (16)	0.7372 (16)	0.041 (4)*	
H6	−0.0302 (18)	0.8170 (16)	0.8721 (16)	0.042 (4)*	
H11	0.0326 (17)	0.3519 (16)	0.6527 (16)	0.040 (4)*	
H10	−0.1435 (19)	0.5362 (16)	0.6423 (16)	0.043 (4)*	
H5	0.1505 (17)	0.7871 (15)	0.9768 (15)	0.039 (4)*	
H23	0.7990 (18)	0.2882 (16)	0.5084 (17)	0.043 (4)*	
H15	0.3788 (18)	0.3804 (17)	0.5071 (16)	0.043 (4)*	
H25	0.819 (2)	0.0269 (18)	0.8685 (18)	0.055 (5)*	
H19	0.255 (2)	−0.0233 (19)	0.7089 (19)	0.057 (5)*	
H16	0.431 (2)	0.3421 (19)	0.292 (2)	0.059 (5)*	
H24	0.923 (2)	0.1070 (19)	0.6230 (19)	0.060 (5)*	
H17	0.389 (2)	0.124 (2)	0.282 (2)	0.071 (6)*	
H18	0.308 (2)	−0.061 (2)	0.494 (2)	0.077 (6)*	

### Atomic displacement parameters (Å^2^)

**Table:**

	*U*^11^	*U*^22^	*U*^33^	*U*^12^	*U*^13^	*U*^23^
O2	0.0438 (6)	0.0805 (8)	0.0329 (5)	0.0227 (5)	−0.0224 (4)	−0.0272 (5)
O1	0.0768 (7)	0.0321 (5)	0.0347 (5)	0.0000 (5)	−0.0287 (5)	−0.0006 (4)
C21	0.0289 (6)	0.0295 (6)	0.0266 (5)	0.0019 (4)	−0.0131 (5)	−0.0093 (4)
C8	0.0246 (5)	0.0314 (6)	0.0226 (5)	−0.0002 (4)	−0.0083 (4)	−0.0063 (4)
C3	0.0235 (5)	0.0331 (6)	0.0239 (5)	0.0006 (4)	−0.0086 (4)	−0.0081 (4)
C1	0.0299 (6)	0.0307 (6)	0.0223 (5)	0.0015 (4)	−0.0115 (4)	−0.0061 (4)
C12	0.0272 (5)	0.0307 (6)	0.0224 (5)	−0.0006 (4)	−0.0095 (4)	−0.0059 (4)
C7	0.0266 (5)	0.0329 (6)	0.0245 (5)	0.0019 (5)	−0.0070 (4)	−0.0051 (5)
C13	0.0349 (6)	0.0282 (6)	0.0279 (6)	−0.0007 (5)	−0.0148 (5)	−0.0052 (5)
C2	0.0275 (5)	0.0345 (6)	0.0235 (5)	0.0030 (5)	−0.0108 (4)	−0.0088 (4)
C22	0.0332 (6)	0.0300 (6)	0.0281 (6)	−0.0011 (5)	−0.0131 (5)	−0.0063 (5)
C14	0.0320 (6)	0.0290 (6)	0.0304 (6)	0.0035 (5)	−0.0167 (5)	−0.0095 (5)
C4	0.0304 (6)	0.0386 (7)	0.0284 (6)	−0.0030 (5)	−0.0109 (5)	−0.0116 (5)
C20	0.0306 (6)	0.0384 (6)	0.0261 (6)	0.0046 (5)	−0.0137 (5)	−0.0103 (5)
C9	0.0264 (6)	0.0442 (7)	0.0290 (6)	0.0073 (5)	−0.0106 (5)	−0.0060 (5)
C23	0.0363 (7)	0.0428 (7)	0.0301 (6)	−0.0041 (5)	−0.0082 (5)	−0.0144 (5)
C11	0.0326 (6)	0.0394 (7)	0.0277 (6)	−0.0023 (5)	−0.0140 (5)	−0.0086 (5)
C5	0.0403 (7)	0.0340 (6)	0.0335 (6)	−0.0023 (5)	−0.0093 (5)	−0.0153 (5)
C6	0.0347 (6)	0.0319 (6)	0.0325 (6)	0.0048 (5)	−0.0073 (5)	−0.0090 (5)
C15	0.0361 (6)	0.0360 (7)	0.0304 (6)	0.0022 (5)	−0.0138 (5)	−0.0083 (5)
C26	0.0404 (7)	0.0374 (7)	0.0309 (6)	0.0079 (5)	−0.0186 (5)	−0.0102 (5)
C10	0.0272 (6)	0.0519 (8)	0.0301 (6)	0.0009 (5)	−0.0152 (5)	−0.0078 (5)
C24	0.0375 (7)	0.0510 (8)	0.0490 (8)	0.0117 (6)	−0.0163 (6)	−0.0297 (7)
C16	0.0385 (7)	0.0623 (9)	0.0288 (6)	0.0115 (7)	−0.0134 (6)	−0.0124 (6)
C19	0.0616 (9)	0.0323 (7)	0.0502 (8)	0.0017 (6)	−0.0318 (7)	−0.0136 (6)
C25	0.0490 (8)	0.0418 (7)	0.0489 (8)	0.0193 (6)	−0.0280 (7)	−0.0195 (6)
C17	0.0667 (10)	0.0746 (11)	0.0499 (9)	0.0307 (9)	−0.0372 (8)	−0.0401 (9)
C18	0.0911 (14)	0.0492 (9)	0.0738 (12)	0.0163 (9)	−0.0525 (11)	−0.0368 (9)

### Geometric parameters (Å, º)

**Table:**

O2—C20	1.2209 (14)	C9—C10	1.377 (2)
O1—C13	1.2179 (15)	C9—H9	0.984 (16)
C21—C22	1.3927 (16)	C23—C24	1.384 (2)
C21—C20	1.4912 (17)	C23—H23	0.952 (16)
C21—C26	1.3913 (17)	C11—C10	1.4133 (19)
C8—C3	1.4101 (15)	C11—H11	1.001 (15)
C8—C12	1.4118 (16)	C5—C6	1.3740 (19)
C8—C7	1.3888 (17)	C5—H5	0.954 (15)
C3—C2	1.4722 (16)	C6—H6	0.988 (16)
C3—C4	1.3779 (17)	C15—C16	1.3915 (19)
C1—C12	1.4697 (16)	C15—H15	0.986 (16)
C1—C13	1.4882 (17)	C26—C25	1.3825 (19)
C1—C2	1.3784 (16)	C26—H26	1.000 (15)
C12—C11	1.3795 (16)	C10—H10	0.993 (16)
C7—C9	1.4225 (17)	C24—C25	1.383 (2)
C7—C6	1.4187 (18)	C24—H24	0.97 (2)
C13—C14	1.4877 (16)	C16—C17	1.373 (2)
C2—C20	1.4854 (16)	C16—H16	0.954 (19)
C22—C23	1.3841 (18)	C19—C18	1.381 (2)
C22—H22	0.985 (15)	C19—H19	0.962 (19)
C14—C15	1.3913 (17)	C25—H25	0.945 (18)
C14—C19	1.3912 (18)	C17—C18	1.374 (3)
C4—C5	1.4163 (19)	C17—H17	0.99 (2)
C4—H4	0.966 (15)	C18—H18	0.96 (2)
			
C22—C21—C20	121.34 (11)	C22—C23—H23	118.1 (10)
C26—C21—C22	119.43 (11)	C24—C23—C22	119.88 (12)
C26—C21—C20	119.18 (11)	C24—C23—H23	122.0 (10)
C3—C8—C12	110.88 (10)	C12—C11—C10	118.34 (12)
C7—C8—C3	124.61 (11)	C12—C11—H11	120.7 (9)
C7—C8—C12	124.42 (11)	C10—C11—H11	121.0 (9)
C8—C3—C2	105.75 (10)	C4—C5—H5	117.5 (9)
C4—C3—C8	118.49 (11)	C6—C5—C4	122.90 (12)
C4—C3—C2	135.74 (11)	C6—C5—H5	119.6 (9)
C12—C1—C13	125.18 (10)	C7—C6—H6	118.6 (9)
C2—C1—C12	108.69 (10)	C5—C6—C7	120.21 (12)
C2—C1—C13	125.37 (11)	C5—C6—H6	121.2 (9)
C8—C12—C1	105.88 (10)	C14—C15—C16	120.18 (13)
C11—C12—C8	118.43 (11)	C14—C15—H15	119.9 (9)
C11—C12—C1	135.51 (11)	C16—C15—H15	120.0 (9)
C8—C7—C9	115.99 (11)	C21—C26—H26	119.2 (9)
C8—C7—C6	115.79 (11)	C25—C26—C21	119.86 (12)
C6—C7—C9	128.18 (11)	C25—C26—H26	120.9 (9)
O1—C13—C1	119.54 (11)	C9—C10—C11	122.70 (11)
O1—C13—C14	121.09 (11)	C9—C10—H10	119.0 (9)
C14—C13—C1	119.36 (10)	C11—C10—H10	118.3 (9)
C3—C2—C20	123.98 (10)	C23—C24—H24	120.3 (11)
C1—C2—C3	108.80 (10)	C25—C24—C23	119.95 (13)
C1—C2—C20	127.13 (11)	C25—C24—H24	119.7 (11)
C21—C22—H22	119.0 (8)	C15—C16—H16	119.4 (11)
C23—C22—C21	120.36 (12)	C17—C16—C15	120.08 (15)
C23—C22—H22	120.6 (8)	C17—C16—H16	120.6 (11)
C15—C14—C13	122.05 (11)	C14—C19—H19	116.6 (11)
C19—C14—C13	118.87 (11)	C18—C19—C14	120.02 (15)
C19—C14—C15	119.01 (12)	C18—C19—H19	123.3 (11)
C3—C4—C5	117.97 (11)	C26—C25—C24	120.50 (13)
C3—C4—H4	121.3 (9)	C26—C25—H25	119.2 (11)
C5—C4—H4	120.6 (9)	C24—C25—H25	120.3 (11)
O2—C20—C21	120.85 (11)	C16—C17—C18	120.00 (14)
O2—C20—C2	120.00 (11)	C16—C17—H17	120.5 (12)
C2—C20—C21	119.11 (10)	C18—C17—H17	119.4 (12)
C7—C9—H9	119.8 (9)	C19—C18—H18	117.3 (12)
C10—C9—C7	120.12 (12)	C17—C18—C19	120.70 (15)
C10—C9—H9	120.1 (9)	C17—C18—H18	121.9 (12)
			
O1—C13—C14—C15	−158.47 (13)	C7—C8—C12—C11	−0.42 (17)
O1—C13—C14—C19	18.35 (19)	C7—C9—C10—C11	−0.05 (19)
C21—C22—C23—C24	1.11 (19)	C13—C1—C12—C8	170.16 (10)
C21—C26—C25—C24	1.1 (2)	C13—C1—C12—C11	−4.8 (2)
C8—C3—C2—C1	−0.11 (13)	C13—C1—C2—C3	−170.17 (10)
C8—C3—C2—C20	−177.02 (11)	C13—C1—C2—C20	6.62 (19)
C8—C3—C4—C5	1.23 (17)	C13—C14—C15—C16	176.96 (12)
C8—C12—C11—C10	0.71 (17)	C13—C14—C19—C18	−177.84 (14)
C8—C7—C9—C10	0.35 (17)	C2—C3—C4—C5	−177.04 (13)
C8—C7—C6—C5	−0.36 (17)	C2—C1—C12—C8	−0.25 (13)
C3—C8—C12—C1	0.19 (13)	C2—C1—C12—C11	−175.18 (13)
C3—C8—C12—C11	176.14 (10)	C2—C1—C13—O1	42.45 (18)
C3—C8—C7—C9	−176.22 (11)	C2—C1—C13—C14	−138.63 (12)
C3—C8—C7—C6	1.77 (17)	C22—C21—C20—O2	−149.50 (13)
C3—C2—C20—O2	38.50 (18)	C22—C21—C20—C2	28.47 (17)
C3—C2—C20—C21	−139.48 (12)	C22—C21—C26—C25	−0.45 (19)
C3—C4—C5—C6	0.07 (19)	C22—C23—C24—C25	−0.5 (2)
C1—C12—C11—C10	175.16 (12)	C14—C15—C16—C17	0.4 (2)
C1—C13—C14—C15	22.63 (17)	C14—C19—C18—C17	1.2 (3)
C1—C13—C14—C19	−160.54 (12)	C4—C3—C2—C1	178.31 (13)
C1—C2—C20—O2	−137.84 (14)	C4—C3—C2—C20	1.4 (2)
C1—C2—C20—C21	44.18 (18)	C4—C5—C6—C7	−0.5 (2)
C12—C8—C3—C2	−0.06 (13)	C20—C21—C22—C23	176.88 (11)
C12—C8—C3—C4	−178.80 (10)	C20—C21—C26—C25	−178.03 (12)
C12—C8—C7—C9	−0.13 (17)	C9—C7—C6—C5	177.34 (12)
C12—C8—C7—C6	177.86 (11)	C23—C24—C25—C26	−0.6 (2)
C12—C1—C13—O1	−126.40 (13)	C6—C7—C9—C10	−177.34 (12)
C12—C1—C13—C14	52.52 (16)	C15—C14—C19—C18	−0.9 (2)
C12—C1—C2—C3	0.22 (13)	C15—C16—C17—C18	−0.1 (2)
C12—C1—C2—C20	177.01 (11)	C26—C21—C22—C23	−0.64 (18)
C12—C11—C10—C9	−0.51 (19)	C26—C21—C20—O2	28.03 (18)
C7—C8—C3—C2	176.50 (11)	C26—C21—C20—C2	−154.00 (12)
C7—C8—C3—C4	−2.25 (18)	C16—C17—C18—C19	−0.7 (3)
C7—C8—C12—C1	−176.37 (11)	C19—C14—C15—C16	0.15 (19)
