# Supplementary figures and images for: Crystal structure of 1,2-di­benzoyl­ace­naphthyl­ene
Source: Acta Crystallogr E Crystallogr Commun. 2015 Jun 13;71(Pt 7):o487–8. doi: 10.1107/S2056989015011160 (PMC4518958; doi:10.1107/S2056989015011160)

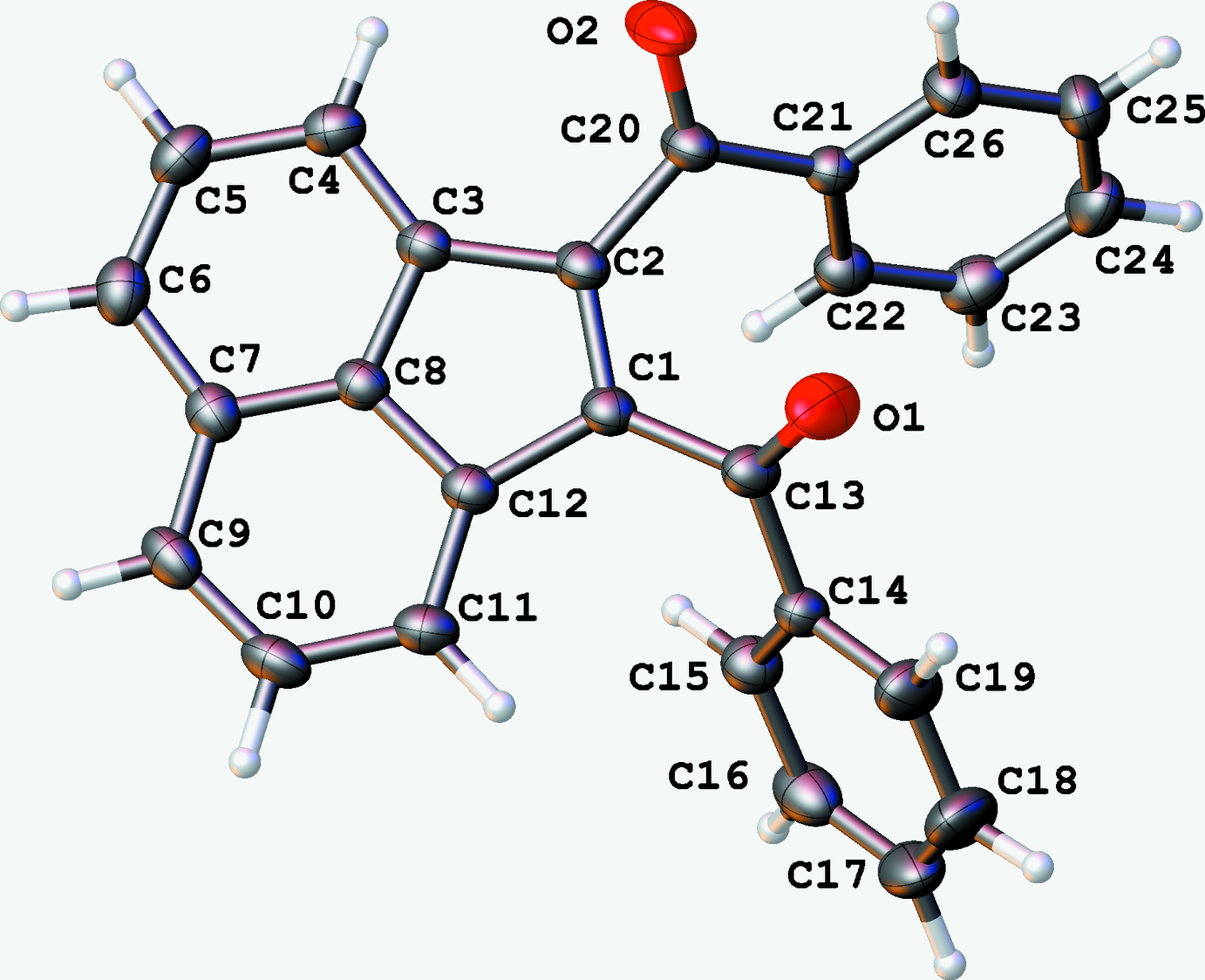

Supplement: Supplementary file 6 [file e-71-0o487-fig1.tif]

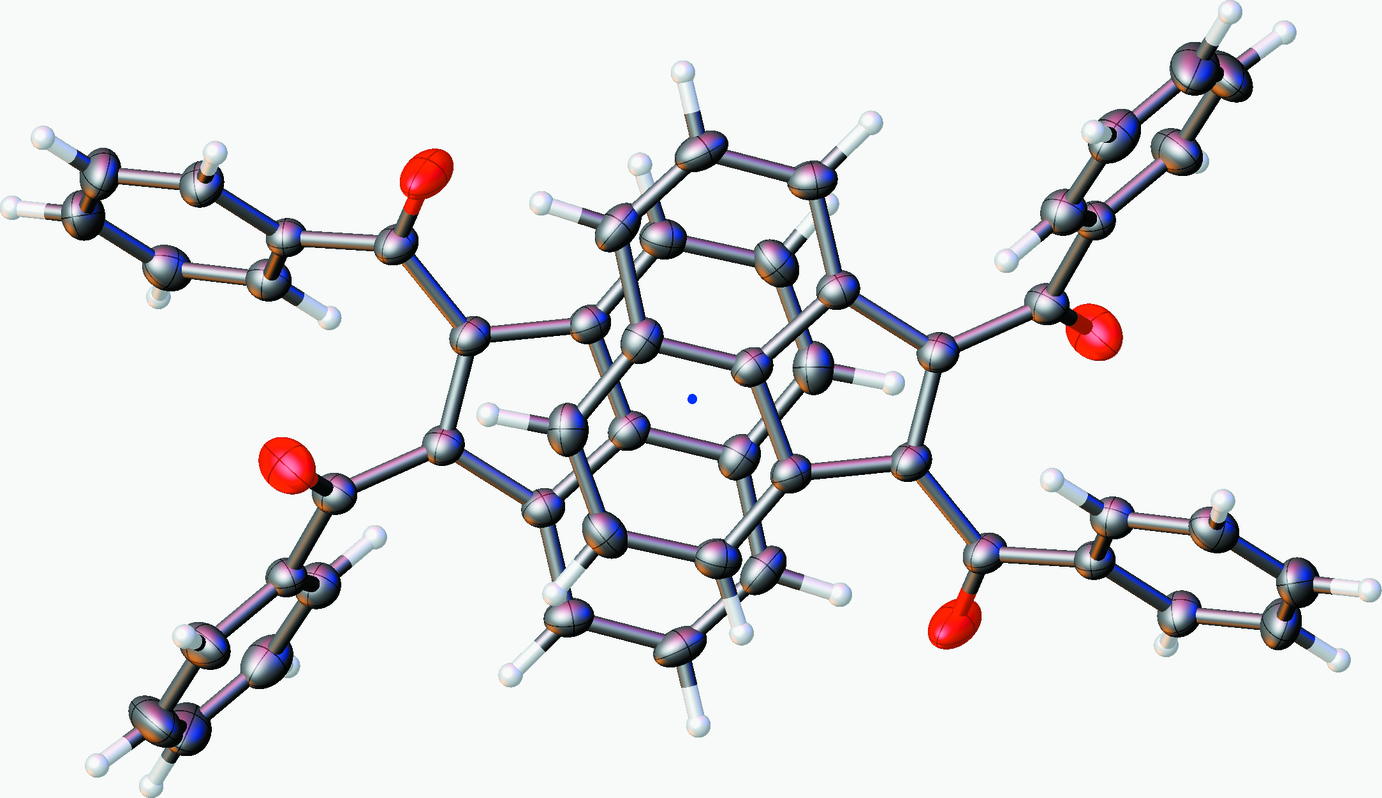

Supplement: Supplementary file 7 [file e-71-0o487-fig2.tif]
